# From emergence to amplification: an analysis of lifecycle models to address health mis-disinformation in the digital environment

**DOI:** 10.3389/fmed.2026.1814102

**Published:** 2026-08-04

**Authors:** Ben Sheppard, Linda Durnell, Amy J. Haufler

**Affiliations:** 1 Applied Physics Laboratory, Johns Hopkins University, Laurel, MD, United States; 2 Whiting School of Engineering and Johns Hopkins School of Medicine, Johns Hopkins University, Baltimore, MD, United States

**Keywords:** artificial intelligence, disinformation, health, interventions, misinformation, technology, risk perception

## Abstract

Health misinformation and disinformation (mis-disinformation) on social media presents a growing threat to individual and population health, societal resilience, and national security. While social media enables the rapid dissemination of health information, it also facilitates the spread of health mis-disinformation, a challenge further compounded by foreign influence campaigns, AI-generated content, and divergent regulatory environments. Effective interventions require tailoring to local socio-cultural and geo-political contexts. This article proposes a lifecycle model for health professionals that conceptualizes how content creation, dissemination, exposure, belief formation, and behavioral outcomes interact and can be targeted through strategic interventions to improve health outcomes and mitigate adverse behavioral effects. To achieve this, the article characterizes the challenges posed by the current and emerging health information environment; identifies and evaluates mis-disinformation lifecycle models in order to strengthen the existing knowledge base; and assesses the current state of knowledge and gaps on comparing intervention strategies to elicit desired behavioral responses, with an emphasis on individual approaches (e.g., debunking, media literacy, fact checking). A review of literature (2020–2025) identified 13 cross-comparative intervention studies which focused on key findings. Four lifecycle models were identified and assessed against six criteria derived from the lifecycle literature to identify the most suitable framework for adaptation in the public health domain. Kruijver et al.'s C5 Interaction Model emerged as the framework that satisfied the greatest number of criteria and was selected for adaptation. The model was extended to account for diverse socio-political and information environments, emerging technological interventions, and the distinct challenges posed by both mis-disinformation. Adaptation involved integrating concepts from risk perception, the Social Amplification of Risk Framework, and Social Judgment Theory, alongside health-specific examples to enhance relevance and practical applicability. To help translate the insights gained to strategy, we also convey the information in an *Integrated Framework for Managing Health Mis-disinformation*. By linking the evolution of health mis-disinformation to targeted interventions, the model and framework provide a foundation for promoting healthier behaviors and mitigating the adverse effects of misleading health information across diverse socio-demographic and cultural settings to improve health outcomes.

## 1 Introduction

The proliferation of health mis-disinformation poses significant threats to public health outcomes and individual well-being. In an era of unprecedented information accessibility and change, the healthcare sector faces significant challenges from the spread of false and misleading health-related information while also developing strategies to capitalize upon the medium's benefits. With over 4.26 billion social media users worldwide, social media has become predominant source for health information, exchange, and influence—providing both benefits and harms to the public's health (1).

Applied appropriately, social media can promote preventive behaviors, improve social connectedness to improve mental health, and facilitate the timely dissemination of actionable health information from trusted sources, particularly during crises when rapid communication is essential (1–6). However, social media research also demonstrates that health misinformation and disinformation (mis-disinformation) can encourage behaviors that are detrimental to individual and population health, including the rejection of evidence-based interventions, pursuing ineffective treatments, and engaging in harmful health practices. These effects have been observed across multiple health domains, including vaccines, cancer care, nutrition, e-cigarettes, and vaping (7–13). Ultimately, mis-disinformation can mislead the public, erode the effectiveness of health programs, compromise individual and community well-being, resulting in negative to catastrophic health outcomes and economic burdens (14–17).

Social media algorithms that are designed to maximize engagement, can amplify false content (18) and sensational or emotionally charged mis-disinformation over factual content (19). This can result in echo chambers where users are presented with information that reinforces their existing beliefs (20). Bots and trolls are increasingly amplifying health misinformation, often for political or financial gain (21). The rapid growth of AI-generated content is already exacerbating the problem (22), with concerns that by 2035 every “fact” is relativized and open to doubt, and overreliance on AI will exceed our ability to fact check (23).

Health system deficiencies may compound the effects of mis-disinformation. Limited healthcare infrastructure, insufficient medical coverage, weak referral pathways, the absence of national treatment guidelines, disease-related stigma, and prevailing cultural practices can collectively create conditions that facilitate the dissemination and influence of misleading health information (24, 25). For example, in Uganda, women tend not to seek timely breast screening and early-stage cancer treatment due to misinformation and associated women healthcare and disease specific cultural and social mores (26–28).

Foreign actors, particularly from Russia and China, exacerbate the health information challenge. They use disinformation as a tool in orchestrated campaigns to sow doubts and counterfactuals in the U.S. and many European countries (29–31). Russia, for example, has amplified disinformation targeting the U.S. on the efficacy of vaccines (32) and on competing public health narratives to sow societal confusion, discord and undermine trust in public health institutions (11).

There is an urgency and a gap to understand, manage and mitigate the adverse effects of social media-related mis-disinformation in the health domain. The purpose of this paper was to identify, analyze, and extend existing mis-disinformation lifecycle model research to adapt a selected framework for navigating the public health digital information environment. A lifecycle model conceptualizes the evolution of mis-disinformation, from content creation and dissemination through to amplification and impact, while identifying opportunities for intervention to improve health outcomes and reduce harmful behaviors. This work sought to develop an actionable, health-centered mis-disinformation model with defined intervention points to better support health practitioners and researchers to improve health outcomes by countering adverse mis-disinformation. We adopt a holistic approach to improving physical and mental health outcomes that enhance individual and population health, increase acceptance of evidence-based interventions, and reduce engagement in harmful health practices.

For the purposes of this paper, we defined mis-disinformation as false or misleading information encompassing both unintentionally shared content (misinformation) and deliberately deceptive content (disinformation). Our definition is inclusive of how the health policy, national security and research communities have approach the challenges of characterizing the information domain and associated consequences (11, 33–39).

The objectives of this paper are to:

1. Characterize the challenges posed by the current and emerging health information environment.
2. Identify and evaluate mis-disinformation lifecycle models to reveal gaps and opportunities to strengthen the existing knowledge base in the health context.
3. Explore, compare and assess intervention strategies that can be employed to supplement the implementation of the lifecycle model. These are divided into two areas. Research on comparing individual approaches (e.g., debunking, media literacy, fact checking). Second, system level approaches (e.g., national regulations and policies) to help contextualize and supplement individual approaches.
4. Examine and introduce concepts from other disciplines such as social judgement theory, risk perception and the social amplification of risk framework to deepen our understanding of mis-disinformation's potency to change behaviors and potential mitigation measures in the health context.
5. Integrate insights from lifecycle models and intervention analyses to develop a tailored framework specifically designed to address health mis-disinformation.
6. Provide recommendations for future research.

Note: The paper is not a meta- or systematic-analysis or a theory paper; rather it synthesizes elements from the literature to develop a customized lifecycle model for the health context. Computational intervention and information characterizing methods such as machine learning, large language models, or social network analysis are out of scope of this study (40, 41).

## 2 Research methods

### 2.1 Lifecycle models

To identify lifecycle models for examination, a literature search was conducted using the terms “lifecycle” and “disinformation” or “misinformation” across Scopus, PubMed, Google Scholar, and JSTOR. The selection criteria required illustrative models that included (or were close to) modeling the entire lifecycle of how misinformation and/or disinformation originates, propagates, is amplified, and can be mitigated through potential interventions. Cyclical models that had the potential to be adapted or expanded for the health information environment were also assessed.

The search identified four published models that met these criteria, with varying depth and breadth of discussion. The four were Tolz et al's. (42), Donovan's (43), Heuer's (38), Kruijver et al's (44) lifecycle models—each of which will be discussed in turn in Section 3.1. The cyclical and depth of analysis varied while each brought important nuances either directly in the model or the discussion for consideration in this research. For example, while Heuer's illustrative framework is linear, its mapping of interventions to the evolution of misinformation over three stages provides a valuable contribution for customizing a lifecycle model for the health information environment.

Six key criteria were used to analyze and assess the selected models and to guide the development of a customized lifecycle model for the health context, with a focus on behavioral outcomes. These criteria were derived from a synthesis of the lifecycle literature and the requirements for adapting a model to the specific challenges of health mis- and disinformation as defined by the authors. The criteria were:

1. Practical application and identification of end users
2. Narrative flow and amplification across linguistic and cultural boundaries
3. Attitudes, behaviors and measures of effectiveness
4. Recognition of potential biases
5. Inclusion of mis-disinformation
6. Interventions

Outside the four models selected for further review and analysis, the search also revealed notable framework approaches including Scales and Gorman (37) study on medical misinformation frameworks, the U.S. Center for Disease Control's Health Information Management and Alert System (HIMAS) framework (45), Bastani's et al.'s application of a framework to COVID-19 (46), and Yu's et al.'s (47) framework for information correction sharing. Although valuable to the literature and included some analyses on the stages and evolution through which false or misleading information is disseminated, they lacked feedback loops to reflect the cyclical nature of mis-disinformation of how narratives evolve and flow together with the complexities of the information environment.

The CDC's HIMAS model, for example, is more intended for managing, monitoring and correcting health information (45). The four-stage approach of pre-planning; monitoring analysis and insights; evaluation and refinement; actions, while useful for guiding steps to developing an action plan, lacks how mis-disinformation can originate, evolve, and amplify. Similarly, Yu et al.'s box charts on factors that can predict misinformation and correction sharing, while valuable for identifying attributes that may increase the likelihood of sharing misinformation, does not include feedback loops and message evolution. Denniss and Lindberg (21) causes, challenges and outcomes together with prevention strategies in their graphical abstract, and Bastani's et. al.'s (46) conceptual frame both lack feedback loops and evolutions of narratives. The Scales et al. study provides a series of insightful graphics on the levels of influence on health, information environment, information flow dynamics but less so of a cycle with the components combined. Nevertheless, such frameworks do provide useful context and overview of the challenges involved particularly those with a health centric approach as with the CDC's HIMARS and Denniss et al.'s frameworks—with insights that are integrated into the proposed *Integrated Framework for Managing Health Mis-disinformation* chart in Section 5.6.

### 2.2 Interventions literature

Mis-disinformation interventions research has grown extensively in recent years propelled by the study of the COVID-19 pandemic that was referred to by the World Health Organization as an ‘infodemic' (48). Research varies from controlled lab-based studies, assessing the effectiveness of interventions in the real world, to subject matter expert observations (encompassing theoretical or practical insights based on case studies).

Literature review searches were conducted on Scopus from 2020–2025 using the search terms “interventions misinformation comparison”; “misinformation interventions” AND “health”; “misinformation” AND “Interventions”. 2020 was selected as the start year to scope the number of searches to a manageable number and focus on more recent literature. The initial search returned a large number of articles with the majority focusing on individual interventions. A subset of 13 studies was identified that conducted cross-comparative analyses of interventions. For the purposes of this review, the analysis focused on key findings and discussion insights rather than the specific comparative methodologies employed or their statistical efficacy.

The primary objective was to assess the extent of convergence across studies in their conclusions on intervention effectiveness—identifying which interventions were most effective, where findings diverged, and highlighting research limitations. The insights were designed to examine the state of the intervention research from a comparison perspective and adaptability to the customized lifecycle model.

## 3 Key lifecycle models characterizing the information challenge

Following a conceptual overview of a lifecycle model, we reviewed, analyzed and summarized selected mis-disinformation lifecycle models to identify key gaps and opportunities for adaptation to the health information domain. We employed the six criteria listed in Section 2.1 to support the selection and analysis of the lifecycle models.

A mis-disinformation lifecycle model is the production, dissemination, exposure, belief formation, and behavioral response to false or misleading information within a dynamic information environment (42, 44) of evolving narratives (49, 50). Content generation can range from the unintentional sharing of inaccurate information to coordinated campaigns by state or non-state actors seeking to mislead audiences and undermine trust in institutions (30, 31, 34). Exposure to such content and subsequent belief formation are shaped in part by individuals' preexisting attitudes, beliefs, heuristics, and social contexts, influencing the likelihood that information will be accepted, questioned, or rejected (51, 52).

These cognitive and social processes can translate into behavioral outcomes, ranging from acceptance of evidence-based interventions to skepticism or rejection of public health guidance (44). Throughout the lifecycle, interventions such as literacy initiatives, inoculation, debunking, and fact-checking can help build resilience to mis- and disinformation (34, 44). Because narratives are dynamic and may be amplified, attenuated, or reshaped by media, institutions, and broader socio-political environments (53, 54), a lifecycle perspective provides a useful framework for understanding the evolution of mis- and disinformation, its behavioral impact, and identifying opportunities for intervention and varying international contexts.

### 3.1 Model summaries

#### 3.1.1 Disinformation Lifecycle

Tolz et al. (42) *Disinformation Lifecycle* model provides a detail of disinformation's linear trajectories and capacity for constant self-renewal. The approach helped to establish a standard for developing lifecycle models by highlighting the diverse dimensions that influence the evolution of disinformation narratives. It is particularly valuable in illustrating how disinformation may gain or lose its classification over time as it traverses geopolitical and linguacultural boundaries, as well as different media genres. Tolz et al.'s (42) approach also illustrates how disseminators of disinformation can customize their narratives, including adverse health information, according to how they believe the target audience (country, culture, demographic, etc.) may perceive it to maximize efficacy. Based on Russian COVID-19 disinformation research, the model comprises of five disinformation elements to reflect diverse actors involved in creating and disseminating disinformation including narrative shifts and transnational actor collusion.

#### 3.1.2 Lifecycle of a Media Manipulation Campaign

Donovan's (43) *Lifecycle of a Media Manipulation Campaign* model is comprised of five points of action that identifies and documents manipulated content through qualitative and quantitative methods from how an actor seeds disinformation on social media, to responses by the targeted audience followed by the malign actor adjusting their campaign in response to counter measures. Although it lacks amplification and detailed interventions, the stages provide a useful topline overview to discuss how manipulated media information may evolve, and response considerations by industry, activists, and journalists. Stage 5 of *Adjustments by manipulators to new environment* resonates well with Tolz et al.'s recognition of how foreign malign actors may manipulate and evolve their narratives to their target audience for greater efficacy.

#### 3.1.3 Phase Model of Misinformation Interventions

As outlined in the methods section, Heuer's (38) *Phase Model of Misinformation Interventions* adopts a linear rather than cyclical structure and identifies three stages: before misinformation is created (Phase 1), after it is disseminated but not yet detected (Phase 2), and once it has been identified (Phase 3). The distinction between Phases 2 and 3 reflects the reality that much misinformation remains undetected for a period—often 10 to 20 h after publication.

**Figure 1:**
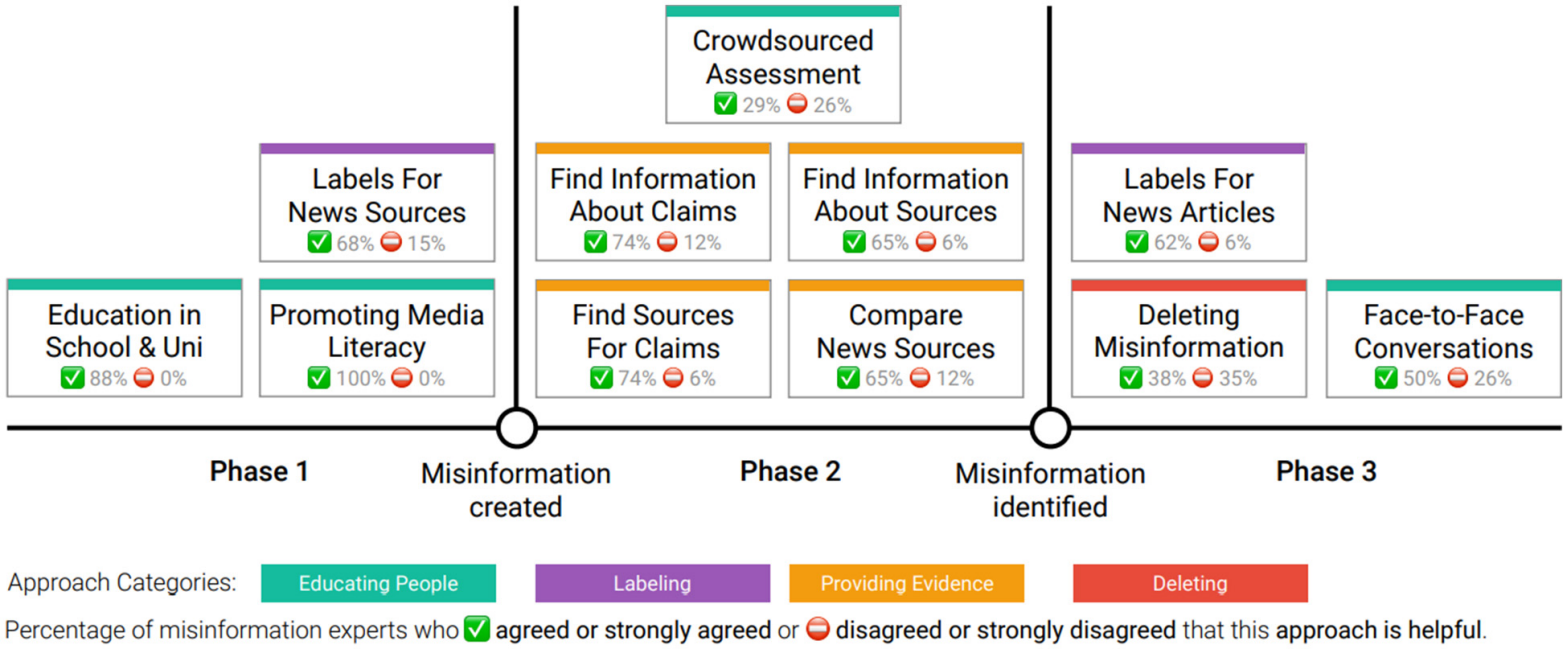
Heuer's *Phase Model of Misinformation Interventions*. Note the three phases: phase 1 (before new misinformation is created), phase 2 (misinformation disseminated but not yet identified), and phase 3 (after misinformation is identified). Interventions are color coded: Educating People (green), Labeling Misinformation (purple), Providing Evidence (orange), and Deleting Misinformation (red). Each intervention includes a percentage of how many misinformation experts agreed or disagreed whether the intervention is helpful. We propose changing misinformation to mis-disinformation for reason discussed in Section 1. *Source*: Heuer et al. (38). Distributed by Association for Computing Machinery under the license CC-BY-SA (https://creativecommons.org/licenses/by-sa/4.0/deed.en).

Although not a full lifecycle model, Heuer's (38) framework contributes valuable insight by integrating subject matter expertise to position 13 interventions across these phases (Figure 1). For these reasons, the approach is listed as one of four main models reviewed. Intervention placement is based on where each is viewed as most appropriate even if an intervention is applicable to multiple stages, for example, media literacy.

While the model is unique with the inclusion of interventions, it lacks many of the nuances found in the other models by Tolz et al. (42) and Kruijver et al. (44), for example, amplification and feedback loops. Despite its linear approach, the suggested interventions to address the evolution of misinformation provides a useful perspective to take into account for future model iterations by outlining the response stages to mis-disinformation.

#### 3.1.4 C5 Interaction Model

**Figure 2:**
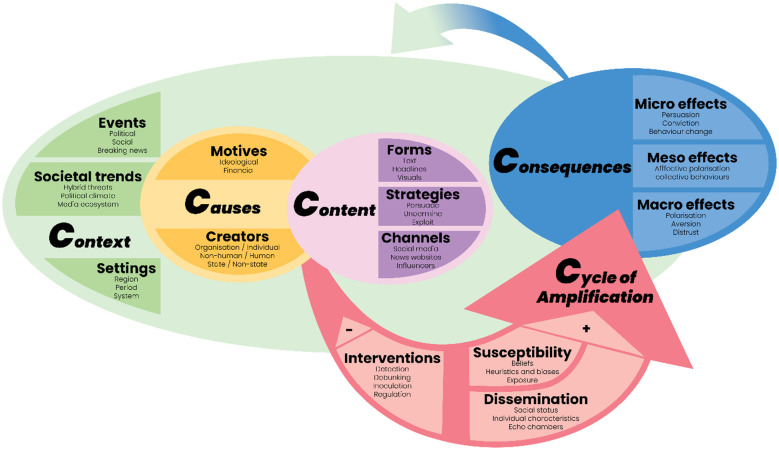
Kruijver et al.'s C5 Interaction Model and the five elements—*Context, Causes, Content, Consequences*, and *Cycle of Amplification*. *Source*: Kruijver et al. (44). Distributed by Springer Nature under the license CC BY NC ND 4.0 (https://creativecommons.org/licenses/by/4.0).

Of the models examined, Kruijver et al.'s (44) disinformation lifecycle model arguably provides one of the most comprehensive attempts to illustrate the flow of disinformation and the various layers involved. The *C5 Interaction Model* is divided into five elements—*Context, Causes, Content, Consequences*, and *Cycle of Amplification*; each of which are then subdivided by two additional layers called factors to capture nuances. Figure 2 illustrates the five elements and their (sub) factors.

While the authors acknowledge that their descriptions are not exhaustive—given the complex, evolving, and context-dependent nature of disinformation—the framework offers a valuable tool for analysts to examine how disinformation develops over time. Particularly relevant to the health context is Kruijver et al. (44) emphasis on “harmful” behaviors that produce a “society-wide impact,” for example, individuals deciding against preventative disease vaccination such as measles regardless of risk to themselves and risk of a community outbreak and associated adverse consequences. This was demonstrated in 2025 where misinformation contributed toward the prevalence of a measles outbreak across multiple U.S. states following some communities refusing to vaccinate their children for measles fearing the combined mumps-measles-rubella (MMR) vaccine causes autism (55). Of the 54 hospitalized patients in West Texas for whom medical records were available, all were either unvaccinated or had an unknown vaccination status (56). Two subsequently died.

### 3.2 Model analysis criteria for developing a health-focused lifecycle model

The following section provides the evaluation of the four selected models against the six key criteria outlined in Section 2.1 to inform the development of a customized lifecycle model for the health context, with an emphasis on behavioral outcomes. The selection criteria were derived from the evaluation of the lifecycle model literature and considerations relevant to model customization. Additional behavioral concepts pertinent to the health area are referenced where relevant to enrich the lifecycle concepts, for example, amplification and the perception of risk.

#### 3.2.1 Practical application

For a model to be effective, it should provide practical utility by offering actionable insights and strategies that address real-world challenges. Practical usage is a theme many models examined. Kruijver et al. (44) stated their research intends to “help guide and inform practitioners on characterizing and handling mis dis mal information”, particularly for those “on the frontline of safety and security work”, while balancing between theory and practice. The frontline point is notable for its applicability to health professionals who interact with patients and may need to navigate health mis-disinformation in their conversations with patients regarding their healthcare treatments and preventative measures. Similarly, Heuer (38) and Kruijver et al. (44) observed that models should help practitioners to make informed decisions so users can better identify misinformation and learn what interventions to leverage. Donovan (43) designed their model to be useful for journalists to “identify, track and expose media manipulation and disinformation.”

#### 3.2.2 Narrative flow and amplification

Narratives are a key component of mis-disinformation and therefore need to be included in a lifecycle model. A narrative can be defined as the representation of an event or a series of events whose stories continuously flows, like a current in a stream (49, 50), and comprised of words, behaviors and actions (57). Narratives are an emergent property from within the cacophony of different ideas, opinions, facts, and information sources that can be amplified by the actions and inactions of the parties involved (50) and result in the propagation of inaccurate information. Narratives that resonate with the targeted community's cultural perceptions and shared beliefs can increase its persuasiveness even if the narrative includes false information—particularly when politically evocative and health issues are combined.

Given the dynamics of narratives flow, lifecycle models need to accommodate that narratives are not static objects, but develop and evolve while claims can be amplified or reduced as they pass through informational, social, or cultural contexts. As Tolz et al. (42) identified, a model needs to reflect the near continuous evolution and fluidity of mis-disinformation flowing across lingual, geopolitical, cultural boundaries and time while also interacting with intervention methods (and their narratives). Tolz et al. (42) illustrated this with bi-directional arrows to depict the dual linear and non-linear dynamism of a cycle without a beginning or end. Tolz et al. (42) also noted that “Rather than traveling as fixed objects, claims are … discursively renegotiated as they journey, acquiring new meanings which are refracted through, and layered over, one another.”

Kruijver et al. (44) built on the amplification theme in the *Cycles of Amplification* element which includes *Susceptibility* (beliefs, heuristics and biases, exposure); *Dissemination* (social status, individual characteristics, echo chambers); and *Interventions* (detection, debunking inoculation, regulation). Deconstructing amplification into these three components provides more insights for targeted intervention strategies. Amplification is discussed more in the results.

#### 3.2.3 Attitudes, behaviors and measures of effectiveness

Ideally an effective model—particularly in the health domain where outcomes serve as a primary benchmark of success—should include guidance on how to evaluate and measure whether and how mis-disinformation is influencing target audience behaviors, and determine to what extent interventions may mitigate adverse behavioral changes. Psychology research shows that attitudes (and by extension influencing attitudes) does not necessarily itself help to understand and predict behaviors (58, 59). However, the models examined contain limited discussion on the role of attitudes and behaviors, and ways to gauge impact. It is important to differentiate between attitudes and behaviors given that it is behaviors that are of interest on how individuals and communities may act or not based on the information they interpret. Tolz et al. (42) infers this point noting their model does not take into account the effects of disinformation on the people subjected to it and associated consequences—viewing this as extraneous to their model. These authors asserted that actual engagement with and experience of mis-disinformation is critical to understanding the mechanisms of mis-disinformation and strategies to counter mis-disinformation. The field of measuring health outcomes and measures of effectiveness is extensive and deserves separate discussion but worth noting key research in this area (60–62).

#### 3.2.4 Recognition of potential biases

Models need to acknowledge potential biases arising from their methodology—whether literature, subject matter experts, or case studies. Ensuring transparency in the strengths, limitations and/or biases in the methodological approach employed is key to the adaptability and customization to local cultures, communities, and information environments. Heuer (38) acknowledged potential biases noting that the cultural and socio-economic backgrounds of the interviewer and the interviewees in their research focused on Western democracies, rather than taking into account Low-Middle Income Countries (LMICs). A case study methodology may offer a valuable avenue for local customization to mitigate Western biases, building on the approach of Tolz et al. (42), who analyzed media articles classified as disinformation to derive lifecycle components. Their study focused on Russian disinformation, examining linguacultural adaptations of narratives and the strategic intent behind tailoring messages for different target audiences. Kruijver et al. (44) briefly discussed a Spanish (Catalonian) case study to illustrate the model's analytical value to reveal the link between disinformation and harmful or criminal behavior.

#### 3.2.5 Inclusion of mis-and/or disinformation

A lifecycle model should encompass misinformation and/or disinformation, whether designed for broad application across information challenges or tailored to specific topical domains. As mentioned in the introduction, we adopted the concept of mis- and disinformation to examine the information landscape from the perspective of social media end users. Examples of how the model can be applied to misinformation and or disinformation are preferable but not a requirement for the analyses applied in this study. Tolz et al. (42) and Kruijver et al. (44) focused on disinformation, Donovan (43) on media manipulation (encapsulating elements for mis-disinformation), while Heuer (38) did not make a strong distinction between mis-disinformation. Kruijver et al. and Tolz et al. both incorporated case examples to demonstrate the development and practical application of their respective lifecycle models.

#### 3.2.6 Interventions

To enhance the practical utility of lifecycle models, particularly for medical professionals and the broader public health community, clear guidance on intervention strategies is recommended given that, as Kruijver et al. (44) noted, those on the “frontline” handle mis-disinformation. Kruijver et al. (44) lists four interventions (detection, debunking, inoculation, regulation) under the “Cycle of Amplification” element that can be expanded upon. Of the models reviewed, only Heuer (38) comprehensively covered this area observing that including interventions is key to enable practitioners to “make informed decisions about which interventions to focus on and how to best combine interventions”. The DISARM framework provides an extensive catalog of interventions for users to counter mis-disinformation (63). Complicating intervention selection are narratives that may be viewed as misleading by one community but reliable and true in another at the same time due to social and lingua-cultural factors as noted by Tolz et al. (42). Therefore, models need to take into account the subjective nature of mis-disinformation and to ensure selected interventions are appropriate to the target audiences social-cultural perceptions.

### 3.3 Model summary

Overall, a combination of Kruijver et al.'s (44) *C5 Interaction Model* with its multiple layers and flows combined with Heuer's (38) *Phase Model of Misinformation Interventions* provides a good basis to combine and adapt for the health environment. A model would also need to include risk analysis elements of risk perception and social amplification of risk that are pertinent to the health arena given how mis-disinformation impacts the acceptability of perceived risks of public health measures. Furthermore, integrating interventions adds value by clarifying which mitigation strategies can be applied, and at what stage on the lifecycle model. Given the critical role of interventions, it is important to closely examine and assess the current state of research in this area which in turn provides a foundation and a menu of options for developing a customized model as discussed in Section 5.

## 4 Cross comparisons interventions review

In the analyses of the lifecycle models, a significant gap in interventions and efficacy was identified. This section reviews the literature on interventions to counter mis-disinformation, outlining the principal approaches across informational, technological, and policy domains which can then be integrated into the customized lifecycle model. It also adopts Roozenbeek and van der Linden's (34) distinction between individual-level and system-level interventions as an organizing framework. The analysis of interventions is based on comparative research to cross compare interventions efficacy (rather than individual intervention research studies) and highlights key strengths, limitations, and gaps—particularly in relation to their applicability to health mis-disinformation in an international context.

### 4.1 State of interventions research

**Figure 3:**
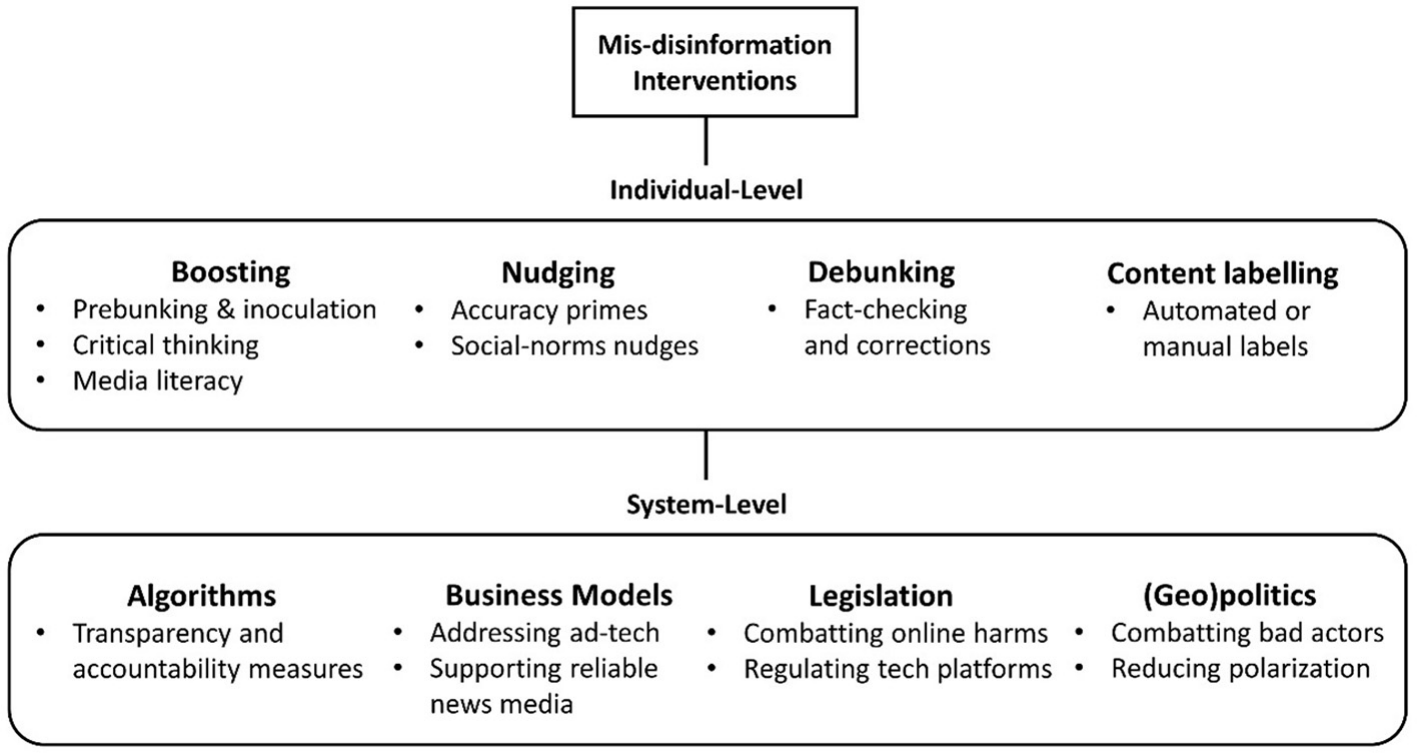
System-level and individual-level mis-disinformation interventions. The original graphic labeled the top box “Misinformation Interventions”. Including disinformation reflects the discussed in Section 1. Table 1 provides a list of definitions for individual level interventions. *Source*: Roozenbeek et al. (64). ©2023 The Author(s). Distributed as a Hogrefe OpenMind article under the license CC-BY-4.0 (https://creativecommons.org/licenses/by/4.0).

The mis-disinformation interventions research has broadly coalesced around a series of intervention terminologies and approaches. Roozenbeek et al. (34) provides a comprehensive overview of the research encompassing information, technology and policy interventions divided into individual-level and system-level interventions. Individual-level interventions are designed to target people's behavior (online sharing habits) or to reduce their susceptibility to misinformation. System-level interventions are intended to place limits on what people can post and share online through legislation and social media company policies, for example. An advantage of adapting Roozenbeek and van der Linden's categorization is their clustering of various individual- and system-level interventions, for example, listing prebunking and inoculation, critical thinking, and media literacy under “Boosting.” This provided a useful starting point and lexicon to use – an approach not often seen in other mis-disinformation research. See Figure 3 for a breakdown of each and the categorization of various interventions. Definitions of the interventions can be found in Table 1. Table 2 summarizes the key intervention studies discussed later.

**Table 1:** Definitions.

Intervention	Definition
Accuracy primes	Prompts encouraging individuals to consider the accuracy of information prior to sharing, improving discernment without direct correction.
Backfire	An intervention correction that unintentionally reinforces a belief in the very misconception it is attempting to correct or heighten the target audience's skepticism of all information they encounter including trusted sources
Content labeling	Automatically or manually labeling social media posts and internet content to signal potential mis-disinformation or provide additional context.
Critical thinking	Encouraging individuals to systematically evaluate information by analyzing evidence, logic, assumptions, and sources to form reasoned judgments.
Debunking	Reactive corrections that refute false claims after exposure by presenting factual information and explaining why the mis-disinformation is incorrect.
Fact checking	The verification of claims by independent or authoritative sources that assess accuracy and publicly label claims as false, misleading, or unsupported.
Media literacy	Skills that enable individuals to analyze, evaluate, share and create social media or internet content, including understanding bias, incentives, and credibility.
Prebunking (Inoculation)	A preventive intervention that exposes individuals to weakened examples of misleading tactics or narratives before exposure, building cognitive resistance to future mis-disinformation.
Social norms nudges	Interventions that leverage perceived social expectations or peer behavior to influence beliefs or information-sharing behavior.

**Table 2:** Summary table of key intervention studies.

Author(s)	Method/Approach (incl. Dates Covered)	Main findings
Blair et al. (80)	SME interviews across Global North and Global South, and a review of 155 misinformation studies	Preferred interventions: platform alterations, media literacy, journalist training; followed by inoculation and debunking; accuracy prompts and credibility labels least favored.
Chan et al. (82)	Meta-analysis (1994–2015)	Debunking weaker when individuals generate reasons supporting initial misinformation.
Gwiazdziński et al. (68)	Review of 75 studies (2013–2021) using PRISMA-ScR. Classified interventions based on an intervention viability assessment score as oppose to measuring efficacy	Media literacy mid-ranked; inoculation, fact checking, and correction lowest; most viable: source rating, trusted sources, UX manipulation, tagging.
Hameleers et al. (78)	Experimental case study of pre-bunking interventions	Interventions may backfire when delivered to individuals not previously exposed to misinformation.
Heley et al. (66)	PRISMA-ScR; 115 publications (2017–2022)	76 positive, 17 null, 68 mixed; no backfire; health literacy & inoculation promising; one-time interventions weaker.
Heuer (38)	Comparative assessment and subject matter expert interviews	Media literacy most effective across misinformation lifecycle followed by education in schools and universities.
Hoes et al. (76)	Online experiment, 6,127 participants (US, Poland, Hong Kong), 2024	Fact-checking, media literacy tips, and news coverage can cause generalized skepticism (backfire-like effects).
Janmohamed et al. (81)	Meta-analysis of 16 COVID-19 studies (33,378 participants, 2020–2021)	Greater engagement increases receptiveness to mitigation guidance; psychological resistance to evidence-based information may weaken an intervention's effectiveness
Kisa et al. (10)	PRISMA-ScR; 256 articles (Dec 2019–Sept 2023)	Effective: digital tech, authoritative communication, fact-checking; no statistical comparisons; focused on interpretive synthesis.
Kozyreva et al. (172)	Review of 81 studies	Found few long-term studies; many show decay of effects; variation in stimuli/outcome measures limits comparability.
Pennycook et al. (73)	Meta-analysis of 20 experiments (2017–2020)	Accuracy prompts replicable and generalizable; reduce sharing of false headlines.
Smith et al. (67)	Review of 50 COVID-19 misinformation studies (2020–2023)	Effective: media literacy, debunking, warning labels, overlays; reduce belief/spread of misinformation.
Whitehead et al. (79)	Review	Interventions not adequately acknowledging scientific uncertainty can backfire; effective: weight-of-evidence, scientific consensus, humor, warnings.

There are other approaches like the DISARM Framework (63) that provided, in addition to a cataloged of interventions, a detailed common language and approach for documenting influence operations and developing a detailed response plan. DISARM is recommended for those looking to employ a set of detailed frameworks.

### 4.2 Individual intervention efficacy

#### 4.2.1 Efficacy

Comparative intervention studies identified moderate to strong efficacy across diverse approaches, with several concluding a low risk of adverse consequences for an intervention to “backfire”. “Backfire” is when an intervention correction unintentionally reinforces a belief in the very misconception it is attempting to correct or heighten the target audience's skepticism of all information they encounter including trusted sources (65). Efficacy concerns the effectiveness of an intervention to have the desired effect. While several studies converged on the effectiveness of specific interventions, others reported divergent findings, reflecting variation in outcomes or interpretation. However, because the methodologies employed in these comparisons were not uniform, the findings cannot be considered direct side-by-side evaluations. Nevertheless, the collective evidence provided useful insights into the relative effectiveness and potential applicability of different interventions to inform a lifecycle model.

Heley et al. (66) examined 115 publications from 2017 to 2022 using the Preferred Reporting Items for Systematic Reviews Extension for Scoping Reviews guideline (PRISMA-ScR). It was found that variation in the efficacy of interventions with 76 reporting a positive correlation, 17 null, and 68 reporting mixed results. Notably none reported a negative ‘backfire' outcome (although other studies found this not to be the case as will be discussed in 5.2.2). Kisa and Kisa (10) who also used PRISMA-ScR in their analysis of 256 articles between December 2019 and September 2023, concluded digital technology; promoting clear, authoritative communication; and implementing fact-checking mechanism were effective. Fact checking is the verification of claims by independent or authoritative sources that assess accuracy and publicly label claims as false, misleading, or unsupported. However, unlike Heley et al., Kisa et al. (10) did not conduct a side-by-side statistical comparison of intervention efficacy. Instead, they synthesized reported observations from individual studies to construct an informative comparison table that highlighted useful features, such as the intended target audience of each intervention.

Several comparative studies consistently highlighted health/media literacy, inoculation, and debunking as particularly effective. Media literacy are skills that enable individuals to better assess the veracity of content. Inoculation intervention exposes individuals to weakened examples of misleading tactics or narratives before exposure, building cognitive resistance to future mis-disinformation. Heley et al. (66) found that health literacy and inoculation interventions show promise although one-time interventions are less likely to alter attitudes. Kisa et al. (10) and Heuer (38) also viewed health literacy as potentially effective. Similarly Smith et al. from their COVID-19 misinformation analysis of 50 studies published between 2020 and 2023 found evidence to show that media literacy tips and debunks (reactive corrections that refute false claims), together with warning labels, and overlays mitigated the spread of or belief in COVID-19 misinformation (67).

Interestingly media literacy / digital literacy was not an intervention that Gwiazdziński et al. (68) identified to be the most effective across the 75 studies reviewed from 2013 to 2021. Media literacy was rated in the middle of the 15 interventions assessed for efficacy, and inoculation, fact checking and correction rated the lowest. Using the PRISMA-ScR methodology, rating the source, message from a trusted organization, UX manipulation, and tagging were rated the most viable (68).

Variations between Gwiazdziński et al. and those of Heley et al., Kisa et al., and Heuer's studies may be in part to the various methodologies employed. Gwiazdziński et al. classified interventions based on an intervention viability assessment score survey to evaluate the possible reach and overall cost of their implementation on social media platforms as oppose to measuring efficacy that Heley et al., Kisa et al., and Heuer examined.

Promising insights on media literacy is significant given that many countries including those in Scandinavia and the Baltics have integrated media literacy into their educational system, partly to counter Russian disinformation targeting their countries (69, 70). Studies such as those by D'Errico et al. highlight how social approaches based on psychology, can strengthen analytical thinking, emotional awareness, and resistance to misinformation among adolescents (71). With health literacy found to be a strong determinant of health (72), emphasis on media literacy may provide significant overall health benefits. There are, however, scalability and practicality challenges outside the education setting. For example, most adults are not currently enrolled in higher or continuing education and may be less inclined to take an in person or online media literacy course for the time and effort required and/or perceived need vs. benefit. Furthermore, cultures in some countries may perceive media literacy efforts as imposing a certain ideological perspective as has occurred in the US. Depending on a country's socio-political environment, media and digital literacy efforts can be delivered through various channels including education (primary, secondary and higher education) via national-local government initiatives or non-profit-civic groups, together with information campaigns for adults.

While a review of comparison studies revealed differing conclusions as to which interventions were shown to be the most effective, an analysis of studies within a single intervention area do suggest some repeatability. Based on a meta-analysis of 20 experiments (2017–2020), Pennycook et al. (73) found that accuracy prompts (nudges) were a replicable and generalizable intervention that increased the quality of news that people shared and reduced the likelihood of sharing false headlines. Psychological inoculation (a proactive “prebunking” strategy that builds cognitive resistance against false information) has been found to be effective as well (74).

#### 4.2.2 Backfire effect

A key issue to consider is whether interventions may cause a “backfire” effect. While Heley (66) and Walter et al. (75) found no “backfire” risk in the studies they reviewed, research conducted by Hoes et al. (76) found that interventions, such as fact-checking, media literacy tips and news coverage of misinformation, may inadvertently condition individuals to “approach all information, whether false or true, with heightened suspicion and skepticism” (76).

Similar findings were reported by van der Meer et al. (77), and Hameleers et al. (78) who observed that delivering interventions to individuals not previously exposed to misinformation may result in stronger negative effects than the positive effects associated with correcting misperceptions. For example, media literacy efforts may, according to Hameleers et al. study, (78) unintentionally undermine people's trust in factually accurate information among those not exposed to mis-disinformation.

In addition, interventions that do not adequately acknowledge scientific uncertainty (e.g., vaccine risks) may backfire too according to a review by Whitehead et al. (79). However, Whitehead et al. (79) added “communicating the weight-of-evidence and scientific consensus around vaccines and related myths, using humor and incorporating warnings about encountering misinformation” was found to be promising (79). This is significant in the health context when addressing heightened perceived risks individuals may hold when public health authorities communicate complex evidence-based science especially when there may be scientific uncertainty in or around the evidence presented.

#### 4.2.3 Subject matter expert evaluations

Another way to assess interventions effectiveness is subject matter expert input. Heuer (38) found that all interviewed subject matter experts identified media literacy as the most appropriate intervention across all stages of the misinformation lifecycle, particularly prior to the creation and dissemination of misinformation. Similarly, education in schools and universities was also found to be popular. Blair et al. (80) who interviewed subject matter experts from the Global North (northern hemisphere) and Global South (southern hemisphere) found that both groups identified platform alterations, media literacy, and journalist training as preferred intervention methods (and least studied) followed by inoculation and debunking (80). Accuracy prompts and credibility labels were the least favored by both groups.

#### 4.2.4 Characteristics of effective interventions

Several key strategic themes may impact the effectiveness of interventions when applied outside controlled laboratory settings and help inform the prioritization of intervention approaches. Passive interventions for which individuals do not need to be a highly motivated to pursue the intervention, e.g., content labeling and source rating, may lend themselves to greater effectiveness and scalability. Passive interventions offer the advantages of scalability and possible automation. For example, AI powered Chatbots (to be discussed in Section 6.2.1) offers promise for empowered patient-clinician engagement especially when AI models significantly improve in accuracy and interaction (68). Interventions that require active individual public engagement on the other hand, such as fact-checking or enrolling in a media literacy course outside of formal education, require individuals being motivated to seek out that intervention.

Second, how much individuals may care and be engage with an issue, such as healthcare, may influence their willingness to update their knowledge overtime (75). Janmohamed et al. (81), in a meta-analysis of 16 COVID-19 studies involving 33,378 individuals (conducted between January 2020 to September 2021) reached a similar conclusion, adding that greater engagement leads to increased receptiveness to mitigation guidance. Conversely psychological resistance to evidence-based information may weaken an intervention's effectiveness. A 2017 meta-analysis by Chan et al. (82) of studies between 1994 and 2015 found that debunking efforts are weakened when recipients of the intervention generate or hold onto their reasons in support of the initial misinformation (82).

This is important as individuals do not objectively assess health information but instead judge it based on one's relative stance on the issues—which in some circumstances may reinforce an existing belief despite its inaccuracy. This resonates with Social Judgment Theory (SJT) that explains how people evaluate persuasive messages based on their preexisting attitudes that will be discussed in Section 5.4.1 for customizing a specific lifecycle model.

### 4.3 Study limitations

There are several caveats for consideration when drawing inferences on the efficacy and appropriate use of interventions from reviewing the research when applying to a lifecycle model—and for future of mis-disinformation research. Most studies were comprised of short-term online experiments, primarily U.S. / Western focused (with limited LMIC coverage), and focused mainly on changing attitudes rather than behaviors. This is important given that interventions ideally need to mitigate adverse behaviors that can undermine individuals' well-being. Heley et al. (66) observed that most studies focused on cognitive outcomes, with fewer assessing behavior or behavioral intentions. Therefore, it remains open as to the effectiveness of interventions researched to elicit desired behavioral changes (66).

The lack of research from non-Western countries is compounded by evidence suggesting interventions tested in the West do not always show encouraging results outside the West (34, 83). Blair et al. (80) brought the research disparity between the Global North and Global South in stark contrast noting that of the 155 misinformation studies they reviewed, over 80% were conducted in one or more Global North countries. This raises questions about the transferability of intervention findings from the Global North to the Global South, given differences in news consumption, media literacy, state capacity, and related factors (80).

The limited availability of longer-term studies evaluating and comparing the efficacy of interventions has been noted by Kisa et al. (10), Roozenbeek et al. (34), Smith et al. (67), and Kozyreva et al. (84). Less than a third of the 81 studies examined by Kozyreva et al. (84) measured long-term effects (ranging from 1 week to 2 months), with many of those identifying a decay in effectiveness (85–87). The lack of real-world simulation, the role of health authorities as trusted sources, and media preference during crises (10, 34) are additional challenges.

Furthermore, research is limited in comparing interventions on different age groups, demographics and cultures who may respond differently to various interventions (88). For example, adults who are younger and female tend to be prone to higher misbeliefs if exposed to health misinformation, according to analysis of 28 studies (89). Variations in health digital literacy can impact the vulnerability of health misinformation with low health literacy increasing vulnerability to misinformation (90, 91), especially for the elderly (92). Some interventions may be less effective for lower educated rural populations (84).

Misinformation study designs and methodologies vary impacting comparability. Kozyreva et al. (84) identified wide variation in both test stimuli used (e.g., news headlines, real-world claims, websites), and measured outcome variables (e.g., belief or credibility ratings, behavioral measures). Blair et al. (80) found in their assessment of 176 studies that while all the intervention types aimed to reduce belief in or sharing of misinformation, outcomes were not measured in the same way, limiting the extent to make direct comparisons (80). Accuracy prompt research tended to measure the sharing of misinformation, whereas debunking and media literacy typically measured beliefs (80).

Further, this paper reports research of various study methodologies to include systematic analysis used in the comparison articles (Heley et al., Kisa et al. and Gwiazdziński et al. using PRISMA-ScR), meta-analysis (Pennycook et al., Janmohamed et al. and Chan et al.), and ethnographic research (Heuer).

Ideally, future studies need to settle on a standardized and coordinated methodological approaches to improve comparisons including agreed upon outcome measures including comparative studies, the inclusion of more public health experts in intervention design, and more video-based and platform-diverse studies (67). For greatest utility to counter mis-disinformation in the health information domain, consistency, reliability and clarity on what works and why is essential.

### 4.4 Systemic interventions: policy and legislation

While individual interventions may be effective in reducing misperceptions, increasing resilience to manipulation, or nudging people into changing their (self-reported) behavior (34), Chater et al. (93) argues that such approaches may be focusing on the symptoms rather than the causes of misinformation. That is to say the institutional structures and information environment gate keepers. Given that health mis-disinformation can undermine the well-being of individuals, communities, and the nation as a whole, national level systemic approaches present opportunities to mitigate the adverse impact of health mis-disinformation and amplify individual intervention efforts. This section summarizes the opportunities and challenges associated with implementing regulation, policy, social media self-regulation, and national education initiatives to more effectively manage and mitigate health mis-disinformation.

At the national level, mis-disinformation is viewed as a public policy issue because it affects public welfare, democratic governance, public health, economic stability, fundamental rights and national security (94). Furthermore, governments are uniquely positioned to respond with policy making measures at their disposal. Given that health is one of several information domains with which national governments are typically concerned, this section takes a more strategic cross domain perspective toward the various approaches countries and regions are taking. In particular, the series of competing pressures and factors governments face when developing and implementing mis-disinformation efforts.

#### 4.4.1 Regulatory divergence and challenges

Complicating lawmakers efforts to regulate mis-disinformation is defining what constitutes mis-disinformation, determining the scope and context when action should be taken, how to evaluate the effectiveness of such efforts, and balancing the freedom of expression with liability in holding social media companies and individuals responsible for disseminating mis-disinformation (95). These policy features have contributed to regulatory divergence across countries and regions—as illustrated by the EU and US approaches. The EU's more proactive content moderation and platform accountability approach contrasts that of the US's emphasis on free speech protections under the First Amendment and Section 230 of the Communications Decency Act (96). The diverse US and EU approaches illustrate the complex interplay between law, ethics, and technology, as regulators seek to counter the adverse effects of mis-disinformation while safeguarding democratic values (96).

At the heart of the EU's approach is the Digital Services Act (DSA), enacted in 2022, which requires large platforms to implement measures to limit the spread of misinformation and hate speech or face penalties. However, the DSA has attracted criticism over free speech concerns and faces practical challenges related to jurisdiction limits and platform compliance (97–99). The EU's broader strategy combines regulation with media literacy initiatives and strategic partnerships to address mis-disinformation while seeking to preserve democratic freedoms (100).

In contrast, the U.S. approach reflects a stronger emphasis on freedom of expression. Regulatory efforts remain constrained by First Amendment protections and Section 230 of the Communications Decency Act, which shields platforms from liability for most user-generated content while encouraging self-regulation (96, 101, 102). Nevertheless, the rollback of several U.S. government initiatives during the mid-2020s—including the closure of the State Department's Global Engagement Center and related programs—illustrates the challenges of sustaining national counter–mis-disinformation efforts in this environment (103–106).

Other countries have encountered similar tensions. The UK's Online Safety Act focuses primarily on illegal and harmful content rather than misinformation (102, 107, 108), while Australia abandoned proposed legislation requiring platforms to regulate misinformation amid concerns over free expression and implementation (109, 110). With social media company self-regulation viewed as insufficient, Australia banned in late 2025 under 16 years old's from accessing online platforms to limit the harmful effects of social media (111). Other countries are likely to pay close attention to whether the desired effect is achieved.

Providing a broader international perspective, Cipers et al. (94) examined counter-disinformation initiatives across 103 countries and 10 regional or international organizations. They found that democracies with high levels of press freedom tend to adopt more holistic approaches, combining legislative, educational, and transparency measures, whereas authoritarian regimes often rely on broad punitive legislation, including the criminalization of misinformation in countries such as China and Russia (94, 102).

#### 4.4.2 Social media companies

Compounding the legislative and regulatory challenges are social medial companies rolling back platform protections to mitigate the distribution of mis-disinformation.

By the mid-2020s, major platforms such as Meta and X had shifted from professional fact-checking to community notes, partly in response to concerns that content moderation infringed upon free expression (112). Where social media companies were once embracing efforts to counter mis-disinformation, in some cases in coordination with governments, they have since taken a more hands-off approach to the issue, and reduced transparency regarding platform algorithms and reduced data availability thereby hindering the ability to study how disinformation is being spread on the platforms, its effects (113), and testing intervention strategies.

Whether social media platforms should serve as primary regulators of mis-disinformation remains contested. Kaushik (114) highlights the tension between the public benefits of fact-checking and content moderation and the commercial incentives of platform business models, which rely heavily on user engagement and advertising revenue (115). Platforms may also be reluctant to assume the role of content arbiters due to concerns over censorship and innovation (116).

Given the complexities and limitations of expecting social media platforms to self-regulate within a fragmented regulatory landscape, a bottom-up approach—engaging non-governmental organizations and civil society actors to inform and empower citizens through media literacy and related initiatives—offers a valuable complementary strategy for enhancing information discernment in the public sphere.

#### 4.4.3 National media and digital literacy education

National education on media literacy (to gather and verify content) and digital literacy (on how to use digital devices while staying safe online) provide policymakers with additional pathways to build societal resilience against mis-disinformation. In particular knowledge to recognize, manage and mitigate engagement with mis-disinformation across health and digital information broadly. Countries and regions that view mis-disinformation as a societal wellness and national security issue have in particular been leaning into national education efforts. For example, Finland and the Baltic countries integrating media literacy into their educational system, partly to counter Russian disinformation targeting their countries (69, 70). The success of national education efforts to counter mis-disinformation may encounter similar challenges posed by policy and regulation: concerns of free speech and how you define mis-disinformation.

Despite the challenges involved, a combination of policy and regulations, encouraging social media companies to self-regulate, and national education measures customized for a country's cultural, regulatory and political environment could serve as a force multiplier when also combined with individual interventions for managing health mis-disinformation. Non-government backed national media and digital literacy efforts are also good to consider such those in the US including non-profit American Association for Retired Persons (AARP) (who are focused on senior citizens) providing internet factchecking tips, and the News Literacy Project that provides educators with media literacy sources to teach. As Olakoyenikan suggested, a more balanced, hybrid regulatory approach could better foster accountability and the preservation of fundamental rights (96). To conceptualize how these elements interact and relate with one another within a broader ecosystem of interventions and mis-disinformation, the next section assesses how they might be integrated into a revised lifecycle model as an organizing framework.

## 5 Results: lifecycle model for health information environment

Drawing on the analysis presented in the preceding sections, including the review of existing lifecycle models, Kruijver et al.'s C5 Interaction Model emerged as the framework that satisfied the greatest number of the six criteria established for an effective health-focused lifecycle model. In particular, the model demonstrated strengths in its practical application for guiding users through stages of disinformation development, its recognition of how narratives flow and become amplified across information environments, and its acknowledgment of the role that heuristics, beliefs, and biases play in shaping perceptions and responses to disinformation.

Although Kruijver et al. (43) focused primarily on disinformation, many of the model's core principles regarding how harmful narratives emerge, spread, and can be mitigated together with its accompanying discussion are also applicable to misinformation more broadly. At the same time, the model retains the distinctive characteristics of disinformation as intentionally deceptive information often embedded within broader hybrid threat strategies. Nevertheless, several refinements are necessary to adapt the C5 model to the health information environment and to fully address the six criteria identified in this review. These included greater consideration of misinformation alongside disinformation, application across linguistic and cultural contexts, incorporation of health-specific behavioral dynamics, and the integration of intervention approaches tailored to the health domain.

**Figure 4:**
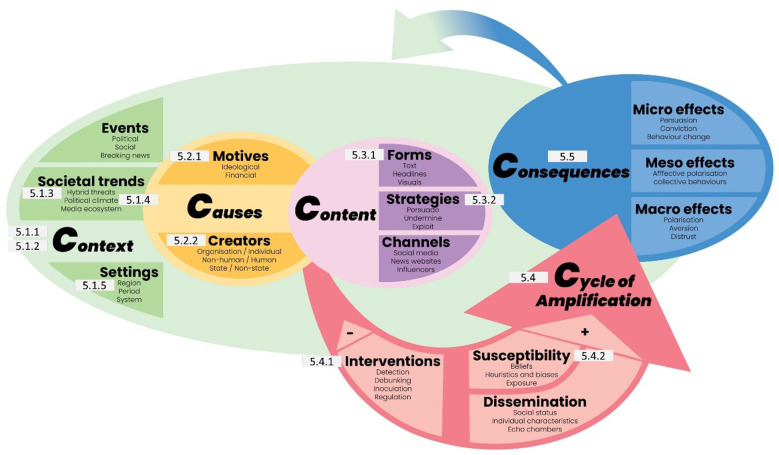
Annotation of Kruijver et al.'s original *C5 Interaction Model* with recommended changes (numbering refers to sub-sections in Section 5). *Source*: Kruijver et al. (44). Distributed by Springer Nature under the license CC BY NC ND 4.0 (https://creativecommons.org/licenses/by/4.0).

Accordingly, the results section is organized around the five core elements of the C5 model—*Context, Causes, Content, Cycles of Amplification*, and *Consequences*. Each element is examined in turn to assess how it can be refined and expanded for application to the health information environment. This process draws on insights from the comparative intervention review, together with introducing new concepts to customize and enrich the model for the health information arena, including risk perception, the Social Amplification of Risk Framework, and Social Judgment Theory. Figure 4 annotates the C5 Interaction Model to illustrate how the subsections in Section 5 correspond to the model's elements and proposed refinements.

The six criteria used to assess and adapt the lifecycle models were as follows:

1. Practical application and identification of end users
2. Narrative flow and amplification across linguistic and cultural boundaries
3. Attitudes, behaviors, and measures of effectiveness
4. Recognition of potential biases
5. Inclusion of mis- and disinformation
6. Integration of interventions

### 5.1 Context

The following recommendations are proposed for adapting the *Context* element to the health information environment. Insert early interventions to reduce individual's susceptibility to mis-disinformation together with assessing the type of policy and regulatory environment for permitting what type of content gets posted online. Under *Societal Trends*, adapt the *Hybrid Threats* sub-factor to take into account health related foreign disinformation, and configure the *Political Climate* sub-factor to recognize the dissemination of non-evidence based public health information. Within *Settings*, strengthen the *Region* subfactor by acknowledging how local cultural, social, and health system factors may influence the local mis-disinformation landscape.

#### 5.1.1 Add early interventions in context

The *C5 Interaction Model* can be strengthened by positioning *Interventions* not only within the *Cycles of Amplification* phase, but also earlier—within the *Context* element—before the creation or detection of mis-disinformation. This follows Heuer's approach of positioning interventions before mis- or disinformation emerges. Doing so allows for proactively shaping narratives, inoculating audiences, and promoting evidence-based health practices, thereby building public resilience before exposure. Users of the model can ask what early intervention methods are already being implemented and should be considered to create some mis-disinformation resilience and mitigate adverse health behavior outcomes?

#### 5.1.2 Add policy and regulatory environment in context

The *Societal Trends* factor should add in a new sub-factor titled ‘*Policy and Regulatory Environment*' to account for the diverse and at times competing policy and regulatory environments across countries that shape what content is posted and disseminated online as discussed in Section 4.4. Divergent social media company approaches may range from robust fact-checking and content removal to softer approaches such as community notes or user-driven flagging.

#### 5.1.3 Hybrid threats

Kruijver et al.'s (44) solid discussion of foreign disinformation and AI exploitation as part of broader hybrid threats can be adapted to the health domain by acknowledging specific instances and approaches of health related foreign influence campaigns. Russia, for example, has exploited the health information environment by amplifying disinformation targeting vaccine efficacy in the U.S. (32) and promoting competing public health narratives intended to generate confusion, social discord, and distrust in public health institutions and institutions broadly in the U.S. and many European countries (29–31).

The hybrid threats factor in the health domain should also account for efforts by states such as Russia to manipulate consumer-based LLMs to produce inaccurate information—a particular concern given that up to one in four users of a leading AI model (ChatGPT) submits a prompt to ChatGPT about healthcare every week (117). Although no documented health-related LLM manipulation campaigns have been identified to date, Russian state-affiliated and state-aligned actors have reportedly incorporated AI into broader influence operations, including attempts to manipulate LLMs into generating false information by promoting malign content for models to ingest and reproduce (118, 119). Similarly, China's influence operations, often mimicking Russian efforts (30), have evolved from the bots, trolls, and click farms of 2019 (30), to using a complex strategy of GenAI, impersonation, profile-hijacking, and coordinated posting (30).

#### 5.1.4 Political climate

Kruijver et al.'s (44) recognition of how political discourse may shape disinformation can be extended to how political environments may support or undermine evidence-based public health guidance. Conflicting messaging may undermine the public's acceptance of information or present information that is factual while in reality it can cause harm. The UK government's mishandling of contaminated beef from Bovine Spongiform Encephalopathy (BSE), or “mad cow disease”, in the 1990s is a salient point. Despite emerging evidence linking contaminated beef to variant Creutzfeldt–Jakob disease (vCJD), a fatal human neurodegenerative condition, the government initially publicly disputed the risks associated with beef consumption despite evidence suggesting otherwise, contributing to widespread public concern and loss of trust (120). Similarly, governments promoting unproven therapies, suppressing data or discouraging vaccinations as occurred in Tanzania and Brazil during COVID-19 (121, 122), can undermine interventions efforts. US efforts to redefine the childhood vaccine schedule in the mid-2020s in the absence of scientific evidence led to divergent and at times conflicting recommendations from the national level to pediatricians and other health-care providers (i.e. family medicine) for parents to navigate (123). Consequently, parents had to navigate these revised recommendations and potentially make non-evidence-based decisions about vaccines and their children's health.

#### 5.1.5 Settings—Region

To broaden the C5 model's international applicability in the health context, users of the model should be cognizant how local cultural, social, and health system factors may influence the local mis-disinformation landscape. In LMICs, for example, health system barriers can exacerbate the information environment with limited health facilities, medical coverage, weak referral systems, and the absence of national guidelines together with stigmatization, familial and religious traditions to navigate (24, 25). The 2026 Ebola outbreak in the Democratic Republic of Congo and Uganda illustrated how the combination of misinformation, cultural perceptions, and limited health access can exacerbate a public health emergency (124).

### 5.2 Causes

#### 5.2.1 Motives

Several aspects of the *Motives* component warrant further emphasis and expansion on, reinforcing points raised both by Kruijver et al. (18) and throughout this paper. For *Financial*, much has been document on how social media platforms, widely attributed as the primary channel of health misinformation, have designed their algorithms to maximize engagement to increase profits with the effect of amplifying false content. Algorithms are designed to amplify sensational or emotionally charged mis-disinformation over factual content (19), creating echo chambers where users are presented with information that reinforces their existing beliefs (20). Industries too are seen to cloud the information environment on trusted sources to help increase sales. Industries such as tanning and tobacco are increasingly using disinformation tactics to present themselves as scientific authorities (21) and may emphasize areas of scientific uncertainty or fund research that supports their interests (21).

Added to this are bots and trolls amplifying health misinformation, often for financial or political gain (21). Section 5.1.3 on Hybrid Threats highlighted several mis- disinformation tactics employed by Russia and China, driven in part by the *Ideological* objectives aimed at weakening the socio-political and economic cohesion of targeted democratic societies. These efforts often seek to further undermine trust in public health institutions and health systems as part of a broader campaign to erode societal cohesion and stability.

#### 5.2.2 Creators

Building on Kruijver et al.'s observation that online personalities strongly shape audience perceptions and behavior, it is important to consider the role “health and wellness” influencers can have within the *Influencers* factor. Some influencers have cultivated alternative online communities and use conspiracy theories, such as anti-vaccine rhetoric, sometimes for the purpose of selling unproven wellness products and services (125, 126). Others have disseminated false narratives that undermine evidence-based health practices, e.g., that sunscreen causes cancer (127). Collectively this may facilitate the spread of mis-disinformation.

### 5.3 Content

#### 5.3.1 Forms

The *Forms* factor, which examined different content formats such as video, text, and images, recognized the role of AI in enabling the large-scale automated production and dissemination of information. In the health context, however, additional consideration should be given to the growing public use of LLMs for health advice and the risk that even well-intentioned health AI chatbots may unintentionally provide inadequate medical guidance. A United Nation's chatbot providing inaccurate health information and Character AI's chatbot promoting eating disorders among teens (128, 129) are just two of multiple health and safety examples.

There are also risks of LLMs unintentionally including mis-disinformation that propagates and reinforces false content if not monitored or ring fenced (as discussed in the Hybrid Threats section), together with the risks of hallucinations by LLMs. Hallucinations are when a LLM perceives patterns or objects that are non-existent, creating non-sensical or inaccurate outputs that often appears plausible.

Over reliance or misuse of platforms like ChatGPT and Claude can have tragic consequences with some individuals using ChatGPT as a therapist taking their own life, for example (130, 131). With OpenAI reporting in early 2026 that one in four of its 800 million regular user base submits a prompt to ChatGPT about healthcare every week (117), the potential for self-harm from AI advice is likely to increase. Given the rapid pace of change in the AI landscape, application of the C5 model to health mis-disinformation will require ongoing adaptation to emerging technological developments and the new challenges they may introduce.

#### 5.3.2 Strategies

Clearer delineation of which components of the model apply specifically to disinformation vs. misinformation would enhance its analytical utility. Because those who disseminate misinformation typically do not act with malign intent, the *Strategies* factors of *Persuade, Undermine*, and *Exploit* are less relevant. By contrast, in the context of disinformation, the *Strategies* dimension could be strengthened by incorporating *Disorientation* as a sub-factor alongside *Persuade, Undermine*, and *Exploit*. Foreign malign influence operations—such as Russian disinformation campaigns—have demonstrated the use of amplifying contradictory or competing narratives on both sides of a public health issue, with the intent to confuse and disorientate the public and erode trust in evidence-based guidance.

### 5.4 Cycles of amplification

There are a several opportunities to integrate additional concepts into Kruijver et al.'s *Cycles of Amplification* component when adapting for the model to the health environment. First, it may be more appropriate to position *Interventions* after the *Susceptibility* and *Dissemination* factors within the *Cycles of Amplification*, as this sequencing allows analysts to first assess a population's vulnerability to mis-disinformation—shaped by underlying beliefs, heuristics, and cognitive biases—which can, in turn, inform the selection and design of the most contextually appropriate interventions. Second, *Interventions* and *Susceptibility* can be customized and expanded upon for health by integrating the concepts of Social Judgment Theory in the *Interventions* factor, and risk perception and the social amplification of risk within the *Susceptibility* factor.

#### 5.4.1 Interventions

There are two elements for improving the *Interventions* factor. First, findings from the mis-disinformation comparative interventions literature on which approaches may serve best in certain circumstances together with their limitations. Second, incorporating SJT as a framework for understanding how individuals' receptivity to interventions may vary based on existing biases.

At the strategic level, insights from the interventions literature suggest that integrating a combination of individual- and system-level approaches into the C5 model's *Interventions* factor is likely to provide the greatest effectiveness. At the individual level, comparative studies identified media and health literacy, inoculation strategies, debunking, fact-checking, and accuracy prompts as promising interventions for reducing misperceptions and strengthening resilience to manipulation, generally with limited evidence of significant “backfire” effects. At the system level, content labels and source ratings resonate with Kruijver et al.'s analysis. While these insights reinforce much of Kruijver et al.'s (44) general intervention recommendations, further enrichment can come from considering limitations of interventions research when applying the model to diverse international and cultural contexts. For example, the limited representation of LMIC and Global South contexts, the lack of longitudinal analyses, and the limited evaluation of sustained behavioral outcomes. Furthermore, users of the model need to customize systematic interventions to the prevailing regulatory environment of the country of concern with the U.S. and E.U, for example having divergent regulatory environments when it comes to regulatory actions such as recommending content moderation and national digital literacy programs as discussed in Section 4.4.

These intervention insights may be further strengthened—and some research limitations partially addressed—by incorporating Social Judgment Theory to assess how receptive target audiences are likely to be to different intervention measures that may vary by different socio-economic, cultural and political dynamics.

Although Social Judgment Theory (SJT), with its focus on biases and heuristics, could logically be positioned within the model's *Susceptibility* factor, it is placed within *Interventions* due to its value in assessing how receptive individuals may be to persuasive intervention messages based on their preexisting attitudes. SJT has three broad categories when assessing individuals' stance (latitudes) of receptiveness to information: Latitude of acceptance, latitude of non-commitment, and latitude of rejection (51). Those with latitudes of acceptance and non-commitment may be more open to interventions. The latitude of rejection refers to those who may object or find unreasonable the information presented and therefore unlikely to be open to changing their perspective given their preferred position (51). The latitude of rejection may be further exacerbated by social media algorithms prioritizing health misinformation content that elicits strong reactions and creates echo chambers to maintain user engagement that also reinforces existing beliefs (19, 20). “Rejection” resonates with the cognitive dissonance concept of discomfort experienced when individuals encounter inconsistencies between one's beliefs and behaviors relative to the information encountered (35, 132). SJT is also useful for Judgmental Anchor: Preferred position on an issue - message acceptance; and Ego Involvement: How important and central an issue is.

The SJT concepts of latitudes of acceptance, rejection, and non-commitment can help explain why some target audiences are more resistant to intervention messages and behavioral change than others. Socio-economic, political, and cultural environments may reinforce particular biases and perspectives, thereby increasing either message acceptance or rejection. For example, MMR and COVID vaccine hesitancy in the U.S. to receptiveness of recommended breast cancer treatments and preventative measures in Uganda as previously discussed.

#### 5.4.2 Susceptibility

Two thematic approaches are recommended for inclusion within the *Beliefs*, and *Heuristics and Biases* subfactors of the *Susceptibility* component when adapting the model to address health mis-disinformation: risk perception and the social amplification of risk framework.

Integrating risk perception characteristics into the model will help users of the lifecycle model to better understand patients' likely perceived risks of recommended health interventions based on the degree of perceived dread and familiarity of the recommendation. Understanding this can then help inform message intervention to meet the concerns of patients. Risk perception research pioneered by Paul Slovic and others (52, 133) on how perceived risk may differ from actual risk, provides a more nuanced understanding of how and why individuals perceive certain risks. Perceived dread and familiarity of a risk may inform individual attitudes and behaviors that, in some cases, may run contrary to scientific evidence. Familiarity (known risk) includes whether it is viewed as observable, whether the effects are delayed, or it is a new risk (134). For example, in the case of mRNA-based COVID-19 vaccines when first authorized for public use, the novelty of the technology, speed of development coupled with concerns about potential delayed adverse effects—including conspiracy theories such as microchip implantation—were amplified by misinformation, despite clear scientific evidence to the contrary (135, 136). Therefore, some communities had a higher perceived dread risk and lower perceived familiarity / comfort with mRNA vaccines leading many to refuse the vaccine. Mapping perceived risk characteristics (degree of dread and familiarity) against actual evidenced-based risk can reveal the degree of divergence to help inform targeted intervention strategies to address areas of heightened public concern (137, 138).

Kruijver's *Cycle of Amplification* concept can also benefit from integrating Kasperson's (54) social amplification of risk framework (SARF) that examines how perceived risks by individuals and communities can be amplified or attenuated based on the psychological, sociological, and cultural perspectives of perceived risks and risk-related behavior (53). The SARF concept was originally designed to help understand how diverse perceived risks from the safety of radon gas, nuclear power stations to drinking water and vaccines, for example, may increase or decrease (53). SARF's key amplification and attenuation stages include how the public processes information, attach social values to information, influence of social groups, traditional and social media, activist organizations, opinion leaders, and public agencies (53).

SARF is well-suited to a health mis-disinformation lifecycle context given that health mis-disinformation centers on how the publics perceive the safety and acceptability of public health and pharmacological interventions which can vary significantly based on the surrounding information, social, cultural and institutional environment. The combination of risk perception and the social amplification of risk framework approach can help guide users of the model on how perceived risks driven by mis-disinformation can be amplified or mitigated, and their cascading consequences.

### 5.5 Consequences

In the health arena the *Consequences* factors of *Micro, Meso* and *Macro* effects can focus on health outcomes and measures of effectiveness (MOE) of the intervention (or adverse impact for mis-disinformation) to assess the impact on health outcomes at the individual, community, and national level. *Micro effects* can include patient- clinician interaction on their health decision making and patient outcomes, for example, parents allowing for their child to receive the MMR vaccine that studies show is safe. The *Meso effects* metrics of collective behaviors can assess MMR community vaccination rates, and during a measles outbreak, adverse consequences such as hospitalization or even death from measles—a preventable disease. At the *Macro effects* level, cascading levels of trust (or mistrust) in institutions and public health entities would likely impact the acceptance (or rejection) of mis-disinformation. It is recommended that subsequent research develops specific measures of effectiveness criteria to assess to what extent intervention strategies may assist with eliciting desired health behavioral outcomes or adverse mis-disinformation undermining key wellness activities.

### 5.6 From insights to strategy: integrated framework for health professionals

To help translate the insights gained to strategy, we convey the information discussed above as the *Integrated Framework for Managing Health Mis-disinformation*. This serves as a summation of the areas discussed throughout that can complement the intervention factors in Kruijver et al.'s original (and now customized) model.

**Figure 5:**
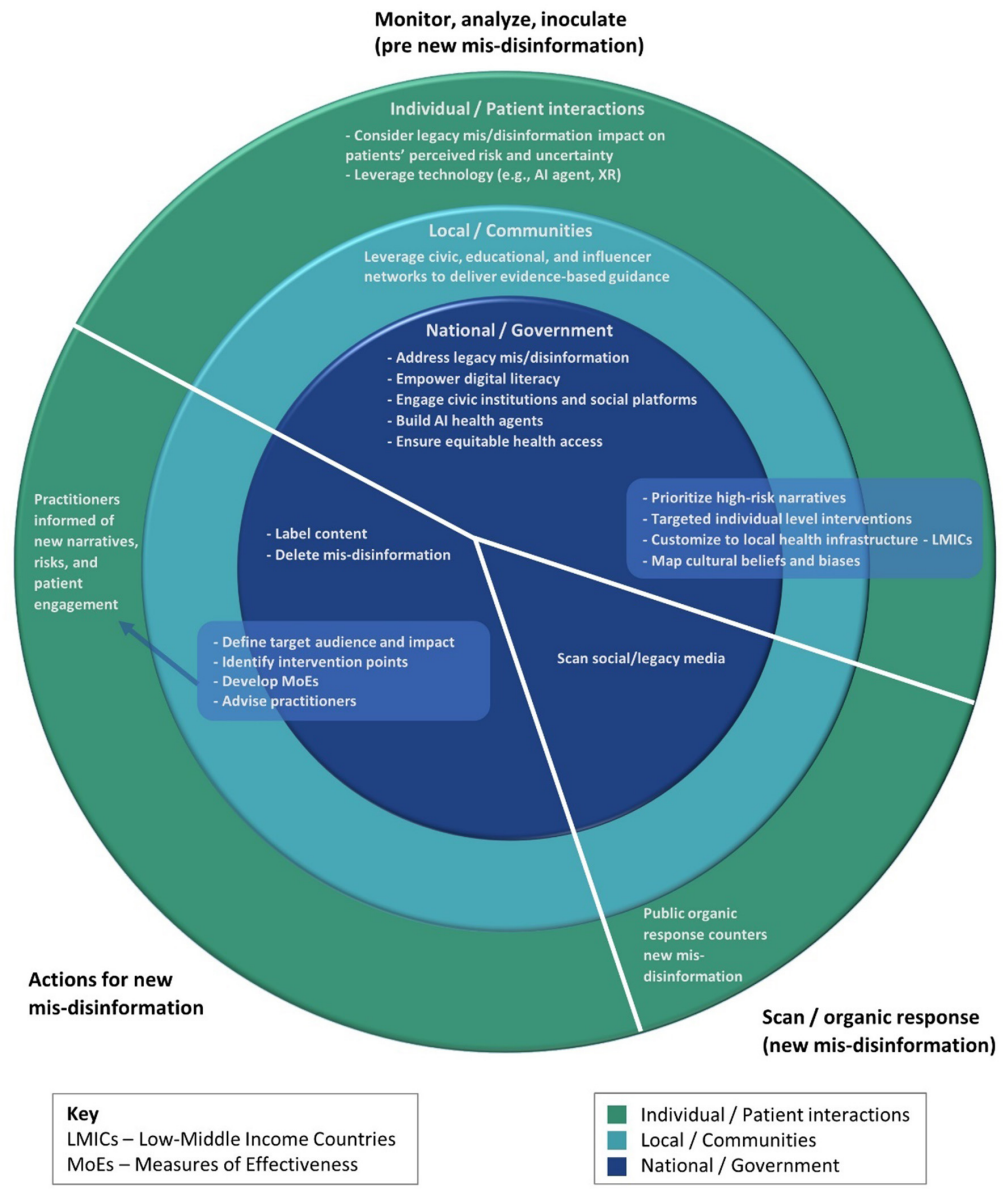
Integrated Framework for Managing Health Mis-disinformation. Depiction of ideal alignment of the national, local and individual-provider levels. In practice, however, these levels are often misaligned, leading to divergent approaches and potential points of friction. Strategies are a summation of insights discussed in the paper. See accompanying Table 3 for detailed breakdown of approaches.

The information is presented in two ways—a table and a chart. Table 3 presents the *Interventions for health professionals* and Figure 5 contains the *Integrated Framework for Managing Health Mis-disinformation* chart. Both integrate the interventions and research findings discussed in the paper by integrating Kruijver et al.'s *Consequences* three factors of different levels of interventions: *Micro* (individual/patient interactions), *Meso* (local/communities), and *Macro* (national/government) engagement levels (rows), and expanding upon Heuer's three phases of interventions approach (columns). The table and chart are designed for health practitioners, community leaders, and national policy developers to gain a holistic understanding of potential approaches and opportunities. They illustrate what interventions to consider before new mis-disinformation is created; automatic interventions to mitigate its initial impacts but before the new mis-disinformation is officially detected; and actions for when new mis-disinformation is officially identified. Together the table and chart are designed to be customizable to multiple countries (high and low-middle income countries). To improve alignment between the national, local and individual-provider levels, each should maintain awareness of how the other may become misaligned to potentially cause points of friction.

**Table 3:** Interventions for health professionals.

Heuer's three phases of misinformation (Figure 1)	Individual level (patient interactions)	Local/community level (leaders and organizations)	National level (government, institutions, national networks)
**Phase 1: Pre-mis-disinformation (Context)**	*Strategic/cross cutting strategies to deliver* (these bullets apply to all three columns) • Media and digital literacy guidance (including in schools, colleges, libraries, non-profit outreach, and information materials in clinician offices)^*^ • Debunking (targeting likely/currently circulating mis-disinformation narratives e.g., vaccine safety-efficacy) • Prebunking and Inoculation (to increase resilience against potential new mis-disinformation) • Encourage critical thinking		
	• Practitioners addresses mis-disinformation concerns with patients as they arise • Practitioners maintain awareness of legacy mis-dis (e.g., MMR vaccines cause autism); and perceived scientific uncertainty and risks of treatments • Patient-clinician interaction to build trust/inform on best information practices (including via AI agent chat bot)	• Leverage civic and education institutions and key influencers to develop/deliver evidence-based guidance • Include NGOs, libraries, religious groups • Develop/distribute public health communication guides (e.g., by research groups, non-profits)	• Identify measures to address legacy mis-disinformation e.g., MMR vaccine/autism. • Address digital divide to improve internet access (digital equity) • Ascertain likely credibility of local institutions to deliver evidence-based guidance • Ascertain foreign malign actor information landscape for possible disinformation • Support development of trusted health AI agents • Labels for news sources • Consider legislation/regulation^*^ • Social media company engagement^*^ (algorithm transparency measures; encourage UX interface mitigations) • Improve local health access to achieve equity
**Phase 2: New Mis-disinformation disseminated—not yet officially detected**	Organic response (automatic public response): • Social-norms nudges and accuracy primes (e.g., from chat forums to trusted media to verify/critique claims) • Verification techniques (individual cross check with trusted sources/compare sources), Crowdsourced assessment		Organic response (national level) • Develop/maintain mis-disinformation alert capability • Scan media (social/legacy); liaise with local health authorities to first detect malign information: Adverse misinformation narrative may first appear in a third country before gaining traction in local country
**Phase 3: New Mis-disinformation detected (cycles of amplification)**	•Deliver authoritative risk communication to address perceived risks: address uncertainty in scientific evidence where appropriate • Encourage fact checking and corrections (debunking)		
	*Practitioner awareness actions* • Become aware of perceived risk characteristic of new mis-disinformation (how the actual risk may differ from the perceived risk as viewed by the patient)	*Local/national actions* • Characterize how the target audience(s)/public(s) may perceive, engage with, and act upon the detected mis-disinformation, taking into account their beliefs, values, and informational contexts • Map likely disconnect between the actual risk (based on science) and perceived risk characteristics of mis-disinformation narrative (degree of dread and familiarity) using the risk perception matrix • Frame how the mis-disinformation may likely be/is being amplified-or attenuated by various digital and legacy media elements (influencers etc.) • Map likely latitudes of acceptance, rejection, non-commitment by likely audience targeted for degree of receptibility of interventions • Develop measures of effectiveness (MOEs) to evaluate the impact of mis-disinformation on health outcomes and to determine interventions effectiveness in mitigating these effects at individual and community levels (e.g., vaccination rates). • Push information to local levels • Develop MOEs to assess impact on health outcomes (e.g., vaccination rates)•Encourage critical thinking, social-norms nudges, accuracy primes	
	*Patient-clinician interactions* • Face-to-face conversations • AI Agent • XR – (for specific health indications-interventions e.g., epilepsy brain surgery)		*National level interactions* • Content labeling (automated or manual) • Delete mis-disinformation

^*^Dependent upon national and local political and cultural acceptance of how much to address mis-disinformation through policy, legislative and regulatory actions.

## 6 Discussion

Success in addressing health mis-disinformation in the digital environment requires sustained commitment, adequate resources, and recognition that this challenge represents a fundamental threat to evidence-based medicine, public health and national security.

The stakes are too high, and the consequences too severe, to treat mis-disinformation less than a critical priority for the healthcare community requiring sustained commitment and adequate resources—not peripheral treatment.

The article set out to examine six main objectives to identify, analyze, and extend existing mis-disinformation lifecycle model research in order to adapt a selected framework for navigating the public health digital information environment. We revisit each of the six main objectives.

Recommendations are integrated throughout the analysis with a separate discussion of technology opportunities.

### 6.1 Strategic insights

We first sought to summarize the challenges posed by the current and emerging health information environment. While social media can disseminate timely and actionable information, it contains a proportion of concerning health content that can undermine people's ability to make informed health decisions. A combination of social media algorithms that raise the profile of contentious information to increase engagement together with foreign actors seeking to intentionally undermine the health information space with disinformation provides a challenging space to address mis-disinformation. The emergence of AI content is likely to exacerbate the challenges further from content generation to dissemination including the reliability of LLMs to provide users with accurate information.

Second, we examined a series of lifecycle models to reveal gaps and opportunities to strengthen the existing knowledge base in the health context. While four were reviewed, Kruijver et al. (44) *C5 Interaction Model* emerged as the most appropriate foundation for further development given its synthesis of academic theory and case studies in both the model and its accompanying discussion. Its flexible structure facilitated the adaptation of the health environment while also making necessary modifications to meet the six prescribed criteria of an ideal lifecycle model. The lifecycle literature is limited on the number of in-depth studies resulting in a limited base to develop a customized model. However, collectively the studies provide a credible foundation to create a tailored version for the health arena.

Modifications made to the *C5 Interaction Model* included integrating concepts from social judgement theory, risk perception and the social amplification of risk framework to enrich the model's structure and applicability to the health context. Doing so enhanced the practical utility of the model by helping users develop an informed understanding of biases and heuristics when to optimize mis-disinformation mitigation approaches.

Adaptation also required the integration of both individual- and system-level interventions, building on approaches identified in the broader lifecycle literature. Comparative intervention studies highlighted several promising strategies for reducing misperceptions and strengthening resilience to manipulation, including media and health literacy, inoculation approaches, debunking, fact-checking, and accuracy prompts. Improving patient health literacy offers considerable potential as it empowers patients to become informed health advocates while maintaining trust in evidence-based medicine.

Despite the plethora of risk intervention research, methodological challenges remain to compare and contrast their effectiveness outside laboratory-controlled environments and in different international contexts. Future research could focus on developing targeted interventions to strengthen patient resilience, conducting longitudinal multi-country studies—including in LMIC settings—using more standardized methodologies, and more fully integrating public health expertise into intervention design and evaluation. Health behavioral outcomes are a key measure of health mis-disinformation intervention effectiveness. It is recommended that future research aims to understand the underlying health attitudes and behaviors to effect positive change in health outcomes.

An additional challenge identified in the interventions literature concerns the scalability, implementation, and public acceptability of both individual- and system-level interventions, which vary across national contexts and are shaped in part by differing socio-political and regulatory environments. For example, intervention measures tend to receive broader institutional support within the European Union and Australia, for example, whereas efforts in the United States have generally been more cautious due to First Amendment protections and the prevailing socio-political climate. Countries facing heightened national security concerns may be more inclined to develop comprehensive counter–mis-disinformation strategies, particularly where hybrid warfare tactics form part of their threat environment. Eastern European countries, for example, have demonstrated increased urgency in responding to Russian disinformation campaigns due to both geographic proximity and broader concerns over hybrid threats.

To synthesize the insights identified throughout the analysis and provide a strategic approach that complements the adapted lifecycle model, we developed the *Integrated Framework for Managing Mis-Disinformation*. The framework provides guidance for implementing interventions at different stages of mis-disinformation development, addressing both supply and demand sides of the ecosystem. This includes foreign malign disinformation that seeks to divide and disorientate societies and to undermine their citizens trust in public health institutions. Defending against targeted health disinformation is a core national security concern. The integrated framework chart can be used to improve alignment between the national, local and individual-provider levels, and where points of friction or misalignment may occur that need to be navigated. Ultimately public concerns surrounding the safety and efficacy of therapeutic treatments and interventions (particular for new and novel drugs) may be better managed and understood by first understanding how they are perceived by individuals (perceived risk)—followed by how mis-disinformation may be amplifying or mitigating adverse narratives as illustrated by the social amplification of risk framework.

### 6.2 Technology opportunities related to intervention

The analysis identified two key emerging technology areas within the health domain that warrant further research as potentially effective intervention strategies to enhance patient–practitioner engagement in addressing mis-disinformation: AI-powered health chatbots and extended reality (XR).

These approaches remain largely underexplored in comparative intervention studies targeting health-related mis-disinformation (139–143). Combined they may provide scalable interventions from credible and trustworthy AI chatbots, to more targeted approaches such as XR to educate patients on specific treatment options and therapies.

#### 6.2.1 AI Chat bots

The emergence of agentic AI, where AI systems including chatbots can autonomously make decisions based on context and act with limited supervision, offers significant potential for customized patient care with the potential to improve health outcomes and counter mis-disinformation and for future research (144). Health AI chatbots are software chat programs that can understand and respond to human input and empathize with the patient with limited to no human decision interventions while providing accurate customized health information.

AI offers scalable fact checking options and health communication options, capable of reaching even billions across social media platforms and enhancing public trust (142, 145, 146). AI factchecking may be particularly effective when directed toward urban populations, according Lee et al. (147) who found enhanced positive attitudes toward COVID-19 vaccination. A 2025 review of 76 AI intervention studies by Saeidnia et al. (148) found that AI can enhance information verification through sophisticated algorithms and natural language processing. Of particular interest is the potential for creating customized LLMs that combat mis-disinformation over a longer period through fact-checking with a high level of accuracy when combined with human oversight and continuous algorithm refinement given the potential scalability and reach of AI (148).

Health professionals could leverage the increasing acceptance of the public consulting AI for health advice (117) develop customized LLMs and user interfaces and supported by appropriate safeguards, to enhance care and present balanced, evidence-based information. Successful implementation could help facilitate informed patient decision-making and help counter harmful mis- and disinformation beliefs that are associated with negative health outcomes.

#### 6.2.2 Leveraging XR (AR – VR) for interventions

A promising yet underexplored and unvalidated clinical–patient engagement intervention in the mis-disinformation literature is the use of Extended Reality (XR) technologies—including virtual reality (VR), Augmented Reality (AR), and Mixed Reality (MR)—to address patient concerns arising from mis-disinformation. VR constitutes the most immersive form of digital experience, creating an entirely artificial, computer-generated environment that completely replaces the user's perception of the physical world (149). AR differs from VR by overlaying digital elements onto the real world rather than replacing it entirely (150). MR represents a more sophisticated integration of digital and physical elements, where virtual objects not only appear within real environments but can interact with physical surfaces and spaces in meaningful ways (151).

Research demonstrates that VR and AR immersive environments may enhance information retention and persuasive impact by eliciting stronger emotional responses than traditional two-dimensional video content (e.g., laptops and mobile devices) (152–154). As a result, these technologies warrant further investigation as an intervention strategy in patient–provider communication.

XR is already well established in healthcare, with validated applications in medical and surgical training (155–158), mental health, anxiety management, autism social skills training, and physical and cognitive rehabilitation (159–164). Patients also benefit from immersive previews of medical procedures and treatment options, including three-dimensional models and interactive demonstrations that can improve understanding and reduce anxiety surrounding care decisions and upcoming surgeries (165–168).

Given the informational advantages of and increasing medical familiarity with XR, its application in the context of health mis-disinformation represents a promising area for future research within broader patient education efforts. XR has already been used to educate epilepsy patients about brain surgery, particularly to address misinformation and concerns surrounding procedural risks and potential long-term behavioral effects (169).

Research could also explore how practitioners and clinicians may benefit from XR technologies through empathy training that immerses them in hypothetical environments that engages them with a patient expressing mis-disinformation concerns on treatment options. Clinicians are already benefitting from empathy training with automated feedback for simulated versions of patient conditions such as aging, visual impairment, or mobility limitations (47).

Despite significant technological advances, XR implementation faces a number of challenges including cost, visual discomfort affecting a subset of users, particularly during extended VR sessions or when experiencing rapid movement in virtual environments (170, 171). Nevertheless, increasing popularity of commercial AR glasses such as Meta-RayBan's further opens the door for exploring XR as an effective use for science communication to elicit desired behavioral and health outcomes (152).

## 7 Conclusion

This study sought to identify and adapt a lifecycle framework for the health information environment to support health practitioners and researchers in managing and mitigating health-related mis-disinformation. A review of the lifecycle literature identified the C5 model as the most suitable foundation for adaptation; however, substantial refinement was required to satisfy the six criteria proposed for an effective health-focused lifecycle model. This process involved synthesizing evidence from comparative studies of individual interventions alongside analyses of systemic interventions, including policy, regulatory, and educational approaches.

The review identified several important gaps in the intervention literature, including the limited availability of longitudinal studies, inconsistent methodologies, and insufficient representation of LMIC and Global South contexts. These limitations raise questions about the transferability of findings derived primarily from Western settings to other socio-cultural environments. To better capture the heuristics, biases, and behavioral dynamics that shape responses to health mis- and disinformation, the adapted model incorporates concepts from risk perception, the Social Amplification of Risk Framework, and Social Judgment Theory. To translate these insights into practice, we developed the Integrated Framework for Managing Health Mis-disinformation, which conceptualizes interventions across the progression of mis- and disinformation narratives, from pre-emergence through detection and response. We also identified AI-powered chatbots and extended reality (XR) as promising areas for future research to enhance patient–practitioner engagement and counter health mis-disinformation.

The proliferation of diverse digital information sources has complicated the public's ability to distinguish evidence-based guidance from misleading content. The resulting erosion of trust in healthcare poses challenges not only for public health but also for societal resilience and preparedness for future crises. It is our hope that the adapted lifecycle model and accompanying framework will provide health professionals with a useful approach for understanding, managing, and mitigating the evolving challenges posed by health mis- and disinformation.
